# Enhancing Mechanical and Antibacterial Performance of Tire Waste/Epoxidized Natural Rubber Blends Using Modified Zinc Oxide–Silica

**DOI:** 10.3390/polym17010109

**Published:** 2025-01-03

**Authors:** Napasorn Kingkohyao, Tanit Boonsiri, Jobish Johns, Raymond Lee Nip, Yeampon Nakaramontri

**Affiliations:** 1 Sustainable Polymer & Innovative Composite Materials Research Group, Department of Chemistry, Faculty of Science, King Mongkut’s University of Technology Thonburi, Bangkok 10140, Thailand; napasorn.king@mail.kmutt.ac.th; 2 Department of Microbiology, Phramongkutklao College of Medicine, Bangkok 10140, Thailand; boonsiri-t@hotmail.com; 3 Department of Physics, Rajarajeswari College of Engineering, Bangalore 560074, India; jobish_johns@rediffmail.com; 4 Global Chemical Co., Ltd., Bangpoo Industrial Estate, Muang 10280, Samutprakarn, Thailand; rlnip@glochem.com

**Keywords:** tire waste, zinc oxide, antibacterial efficiency, epoxidized natural rubber, silica

## Abstract

This study investigates the synergistic effects of incorporating modified zinc oxide–silica (ZnO-SiO_2_) into tire waste (TW) and epoxidized natural rubber (ENR) blends, with a focus on crosslinking dynamics, mechanical reinforcement, and antibacterial activity. The addition of ZnO-SiO_2_ significantly enhanced crosslink density, as evidenced by increased torque and accelerated cure rates. An optimal concentration of 10 phr was found to yield the highest performance. This optimal balance between chemical activation and mechanical reinforcement resulted in exceptional tensile properties, including notable improvements in Young’s modulus, tensile strength, and strain-induced crystallization (SIC). These enhancements were attributed to the strong interactions between ENR molecular chains and SiO_2_ surfaces. However, excessive ZnO-SiO_2_ concentrations caused filler agglomeration, which reduced both mechanical and antibacterial performances. An antibacterial analysis revealed a remarkable 99.9% bacterial reduction at 10 phr ZnO-SiO_2_, attributed to the Zn^2+^ ion release and reactive oxygen species (ROS) generation, with sustained activity even after thermal aging. This durability underscores the composites’ potential for long-term applications. The findings establish ZnO-SiO_2_ as a dual-functional filler that optimizes crosslinking, enhances mechanical properties, and provides durable antibacterial efficiency. These results highlight the potential of TW/ENR blends while offering critical insights into mitigating filler agglomeration to improve overall material performance.

## 1. Introduction

The global demand for sustainable materials has intensified the focus on recycling and upcycling industrial waste, particularly tire waste (TW), which is a major contributor to environmental pollution. Each year, millions of tires are discarded, posing significant ecological and disposal challenges. However, TW, which is rich in rubber, carbon black, and other additives, holds immense potential as a resource for advanced composite materials [1,2]. When blended with polymers such as natural rubber (NR), synthetic rubbers, and modified NR, specifically, epoxidized natural rubber (ENR) [3], TW can be transformed into value-added materials. These composites benefit from the synergistic effects of the active chemicals in TW and the addition of new ingredients, improving the rate of crosslinking, mechanical and dynamic mechanical properties, and thermal stability [4,5]. Due to the unknown ingredients in TW, particularly the mix of hydrophobic and hydrophilic fillers along with various rubber types, epoxidized natural rubber (ENR), derived from renewable NR, offers unique properties, such as enhanced polarity, oil resistance, and compatibility with polar fillers. Additionally, the hydrocarbon chains in ENR can interact physically with the non-polar regions of TW through induced dipole–induced dipole intermolecular forces [6,7]. These attributes make ENR an excellent matrix for enhancing the utility of TW in composite formulations. However, the limited reinforcing ability of residual fillers in TW, such as carbon black (CB), poses a significant challenge. These fillers often exhibit poor dispersion and weak interactions with fresh ENR molecular chains [8,9] due to the presence of embedded rubber molecules from tire preparation. This limitation underscores the need for innovative strategies to enhance filler performance and optimize the properties of TW-based composites.

Recent studies have highlighted the potential of hybrid fillers, such as modified zinc oxide combined with particulate fillers like calcium carbonate (ZnO-CaCO_3_), titanium dioxide (ZnO-TiO_2_), and silica (ZnO-SiO_2_), in addressing these challenges. Previously, ZnO-CaCO_3_ and ZnO-TiO_2_ were used in NR latexes for film and foam applications [10,11,12,13]. These hybrids demonstrated the synergistic effects of ZnO, a vulcanization accelerator with the reinforcing and interfacial adhesion properties of CaCO_3_ and TiO_2_ [14,15], widely used fillers in rubber materials. Modified ZnO hybrids have been shown to enhance crosslink density, mechanical strength, and functional properties, including the antibacterial activity of NR films and foams. The dual functionality of ZnO-CaCO_3_ and ZnO-TiO_2_ also allows them to act as reactive fillers, improving the interactions between rubber chains and promoting the absorption of volatile organic compounds (VOCs) [16]. However, excessive filler concentrations can lead to agglomeration, which diminishes both mechanical performance and antibacterial efficacy. In addition to mechanical reinforcement, antibacterial functionality has become increasingly important for advanced composite materials, especially in healthcare and hygiene applications. ZnO is well documented for its antibacterial properties, primarily attributed to the release of zinc ions (Zn^2+^) and the generation of reactive oxygen species (ROS) [17,18]. A key challenge lies in maintaining antibacterial efficiency during prolonged use and under thermal aging conditions, which is critical for ensuring the reliability of these composites in real-world applications. However, the incorporation of ZnO-CaCO_3_ and ZnO-TiO_2_ into rubber composites using melt blending technology has not been previously examined, particularly regarding the elucidation of the ZnO effect in relation to residual chemicals in TW, such as ZnO, fillers, accelerators, and curing agents. As discussed, the compatibilization of TW with rubbers—especially polar rubbers like ENR—is promising and warrants further investigation, particularly with the use of modified ZnO combined with polar fillers like SiO_2_. The ability of SiO_2_ to form hydrogen bonds with ENR molecular chains enhances the dispersion and distribution of ZnO within the bulk matrix, thereby improving the overall performance of the composite [19].

This study aims to bridge the gap in understanding the multifunctional role of ZnO-SiO_2_ in TW/ENR composites. By systematically evaluating the effects of ZnO-SiO_2_ loading on chemical crosslinking behavior, tensile performance, and antibacterial activity, the study identifies the optimal ZnO-SiO_2_ concentration to achieve a balance between mechanical and bioactive properties. Heat-aging tests further assess the long-term durability of these blends, focusing on the retained activity of ZnO within the TW. The insights gained from this research not only advance the design of high-performance, sustainable materials, but also address critical challenges in recycling TW for high-value applications. The findings hold broad implications for industries seeking to develop eco-friendly, multifunctional materials that combine superior mechanical properties with durable antibacterial functionality.

## 2. Materials and Methods

### 2.1. Materials

Epoxidized natural rubber containing 25 mol% epoxide (ENR25) was obtained from Muang Mai Guthrie Co., Ltd., located in Surat Thani, Thailand. Waste tire rubber (TW) was supplied by Thai Rubb Tech Co., Ltd., based in Bangkok, Thailand, specifically from truck tire waste. The TW was derived from the tire tread of a truck tire after undergoing reclamation processes, which involved the random cleavage of crosslinked rubber chains, both along the rubber main chains and at the -C-S- bonds. With a viscosity of ML (1+4@100 °C) of 52.17, TW may contain an unknown rubber matrix, along with activators such as ZnO and stearic acid, as well as fillers including carbon black (CB), silica (SiO_2_), and/or calcium carbonate (CaCO_3_). Silicon dioxide-modified zinc oxide nanoparticles (ZnO-SiO_2_), with a ZnO:SiO_2_ weight ratio of 90:10 through wet processes, were collaborated and received from Global Chemical Co., Ltd., located in Samut Prakan, Thailand. The ZnO-SiO_2_ nanoparticles possess a surface area of approximately 100 m^2^/g, with the aggregate sizes ranging from 100 to 300 nm and individual particle sizes below 10 nm. Stearic acid and sulfur were procured from Imperial Chemical Co., Ltd., in Pathum Thani, Thailand, while 2,2′-dithiobis(benzothiazole) (MBTS) was supplied by Flexsys Inc., located in Termoli, Italy.

### 2.2. Preparation of TW/ENR Blends Filled with Modified ZnO

The compounding process involved masticating the TW/ENR with modified ZnO in an internal mixer (MX500, Charoen Tut Co., Ltd., Samutprakarn, Thailand) using various ratios of ZnO-SiO_2_ concentrations at 0–20 phr. The mixing conditions were standardized with a temperature of 80 °C, a fill factor of 75%, and a rotor speed of 60 rpm. Initially, the ENR was masticated until its viscosity matched that of TW, after which TW was introduced into the mixer. Subsequently, additional chemicals listed in Table 1, including the ZnO-SiO_2_ with the different concentrations, were incorporated. The total mixing process lasted 8 min, followed by the rolling of the mixture through a two-roll mill (Charoen Tut Co., Ltd., Samutprakarn, Thailand) to enhance dispersion and distribution. Rheometer testing was conducted to determine the production time before the blends were shaped via compression molding at 160 °C. It is important to note that the present formulation was specifically designed to elucidate the synergistic effects between the residual ZnO in TW and the additional ZnO-SiO_2_ in the blends. Consequently, only the key potential chemicals, namely, the co-activator, accelerator, and sulfur curing agent, were included in the formulation.

### 2.3. Cure Characterization

The crosslink propagation of the TW/ENR compounds, along with the details of their chemical crosslinking, was analyzed using a moving die rheometer (MDR3000M, Mon-Tech, Buchen, Germany) operated at 160 °C for 30 min, with a fixed oscillation frequency of 1.66 Hz and an amplitude of 0.5° arc. The key parameters, including scorch time (*T_s_*_1_), cure time (*T*_90_), minimum torque (*M_L_*), maximum torque (*M_H_*), and the torque difference (*M_H_*–*M_L_*), were measured to evaluate the compounds’ curing characteristics and crosslinking efficiency.

### 2.4. Tensile Properties

The tensile properties of TW/ENR blends filled with different concentrations of ZnO-SiO_2_ were evaluated by conducting tensile tests on specimens cut from crosslinked sheets. These sheets were prepared through compression molding into dumbbell-shaped specimens in accordance with ISO 37 Type 2 standards [ISO 37: Rubber, vulcanized or thermoplastic—Determination of tensile stress-strain properties]. The tests were performed using a universal testing machine (Zwick Z 1545, Zwick GmbH & Co. KG, Ulm, Germany) at a crosshead speed of 500 mm/min under room temperature conditions. The tensile strength, elongation at break, and moduli at 100% and 300% elongation were measured and analyzed to assess the mechanical performance of the blends.

### 2.5. Morphologies

The morphologies and elemental compositions of the fractured surfaces of TW/ENR blends were analyzed using scanning electron microscopy (SEM) coupled with energy-dispersive X-ray spectroscopy (EDS). High-resolution images were obtained with an FE-SEM (Nova NanoSEM 450, EDS X Flash 6 series, FEI, Australia) at an accelerating voltage of 10 kV. To prepare the cross-sectional surfaces for analysis, the specimens were cryogenically fractured in liquid nitrogen. The fractured surfaces were then coated with a thin layer of gold to prevent electrical charging during the measurements, ensuring accurate imaging and compositional analysis. In addition, in order to characterize the surface roughness of a wider area, the optical microscope (OM) (Carl Zeiss Microscope GmbH, Oberkochen, Germany) was applied. The blends were quickly cut with a razor blade to create a smooth surface before capturing microscopic photographs. Also, a transmission electron microscope (TEM) (Model JEOL, JEM-1400, Tokyo, Japan), operated at an accelerating voltage of 80 kV, was also used to provide a clearer visualization of the particles. The powder specimens were dispersed in ethanol and sonicated for 30 min, then deposited onto carbon-coated copper grids. The solvent was allowed to evaporate completely before analysis.

### 2.6. Antibacterial Efficiency

The antimicrobial efficiency of the tire waste (TW)/epoxidized natural rubber (ENR) blends was evaluated by quantitatively measuring their ability to inhibit the growth of *Staphylococcus aureus* (*S. aureus*, ATCC 25923) and *Escherichia coli* (*E. coli*, ATCC 25922). Bacterial suspensions were prepared in liquid culture media (Nutrient Broth, NB) with an initial concentration of approximately 10^6^ colony-forming units per milliliter (CFU/mL). A volume of 0.4 mL of each bacterial suspension was placed onto sterilized 5 × 5 cm^2^ samples of the TW/ENR blends, which were kept in a Petri dish. To maintain contact between the bacterial suspension and the surface of the samples while preventing drying, the droplet was covered with a sterilized 4 × 4 cm^2^ polyethylene (PE) film. The samples were then incubated at 37 °C for 24 h. After incubation, both the sample and the PE film were washed with 10 mL of soybean-casein digest medium (SCDLP). Following the washing step, the bacterial suspensions underwent a 10-fold dilution and were plated onto a nutrient agar (NA) medium. After incubation at 37 °C for 24 h, the number of viable bacteria was counted. The percentage reduction in bacterial count (*%R*) was calculated according to Equation (1):

%*R * = [(*A* − *B*)/*A*] × 100 (1)

where *A* is the number of viable bacteria in the treated samples after incubation and *B* is the number of viable bacteria in the control samples after incubation.

## 3. Results and Discussion

### 3.1. Cure Characteristics

**Figure 1:**
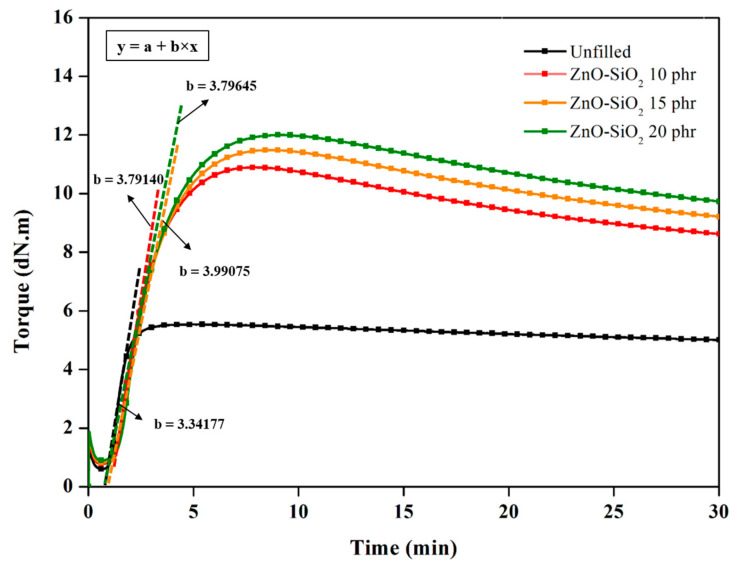
Cure characteristic curves of TW/ENR blend with different modified ZnO-SiO_2_ loading. “Unfilled” refers to the TW:ENR ratio of 50:50 phr without ZnO-SiO_2_.

The cure characteristics of the compounds and vulcanizate composites after exposure to a constant temperature for various durations is illustrated in Figure 1, which depicts the changes in torque as a function of time. When considering the over-cure performance of the composites, reversion curves appear with the addition and increasing concentration of ZnO-SiO_2_. However, the maximum torque tends to increase, which can be attributed to two factors: (i) The higher ZnO content enhances the crosslink density by increasing activator levels in the crosslinking system. (ii) The greater SiO_2_ loading within the bulk TW/ENR matrix improves the reinforcement efficiency, particularly in the ENR phase. Nonetheless, as the degree of polysulfide crosslinking intensifies, thermo-oxidative degradation sets in, leading to a decline in torque after the optimal crosslinking levels are reached [20]. Examining the cure rate slope reveals that a ZnO-SiO_2_ loading of 10 phr results in the steepest slope, indicating the fastest crosslinking rate due to a balanced chemical loading that fulfills multiple roles in activation and reinforcement. However, at concentrations exceeding 10 phr, SiO_2_ agglomeration becomes evident, impacting the overall performance. In addition, the thermal stability of the blends filled with varying concentrations of ZnO-SiO_2_ was evaluated by monitoring the torque as a function of time at a fixed temperature of 160 °C, revealing significant differences. It was observed that the pure TW exhibited a constant torque over time, presenting a characteristic plateau curve. However, when blended with ENR, reversion curves appeared, showing a delayed decrease in torque as the ZnO-SiO_2_ concentration increased. This reversion in ENR is attributed to thermo-oxidative degradation, resulting from the high reactivity of ENR’s epoxide groups with oxygen. Additionally, the increase in polysulfidic bonds leads to the decomposition of sulfur linkages between TW and ENR molecules. As shown in Figure 1, the initiation of reversion was slightly delayed with increasing ZnO-SiO_2_ concentrations. This indicates that the enhanced crosslink density, driven by the higher ZnO-SiO_2_ content, slows down the degradation of rubber chains.

**Figure 2:**
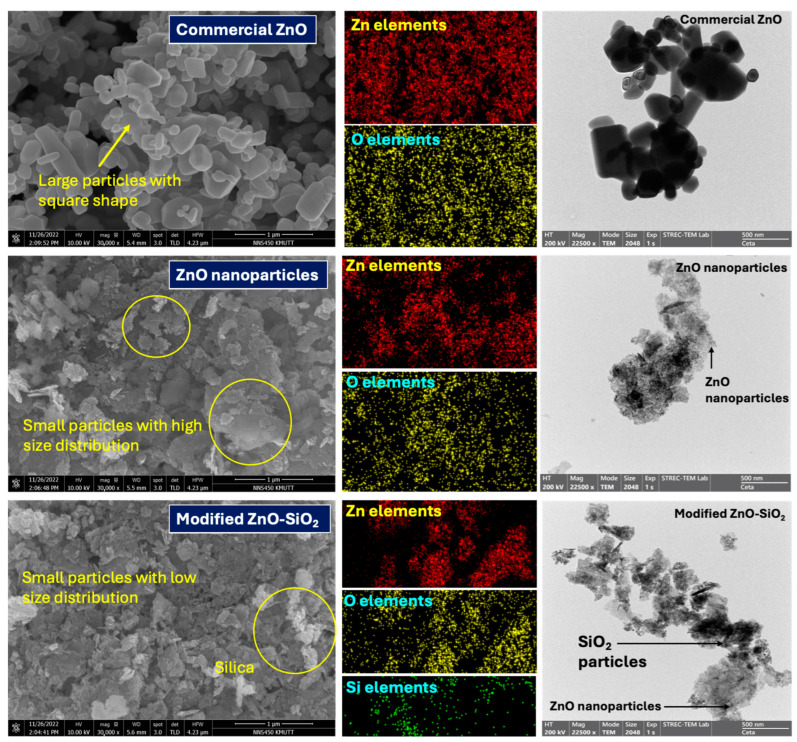
Morphologies of the solely ZnO-SiO_2_, relative to the commercial ZnO.

Figure 1 also illustrates the crosslinking curves, while Table 2 summarizes the minimum torque (*M_L_*), maximum torque (*M_H_*), and the torque difference (*M_H_*–*M_L_*), which corresponds to the estimated crosslink density of the TW/ENR composites. The table additionally provides the scorch time (*T_s_*_1_) and cure time (*T*_90_) of the compounds, highlighting variations in the production time of the composites. The observed increase in *M_H_* with rising ZnO-SiO_2_ concentrations reflects a higher crosslink density, as crosslinking restricts chain mobility and forms chemical bonds between TW/ENR molecules, while also enables the absorption of rubber chains onto the surfaces of ZnO and SiO_2_ particles [21]. Furthermore, Figure 2 shows the morphologies of ZnO-SiO_2_ in comparison to the active ZnO and commercial ZnO white seal. Active ZnO particles are significantly smaller than commercial ZnO. For ZnO-SiO_2_, the chemical absorption of ZnO onto SiO_2_ surfaces results in improved ZnO dispersion due to controlled interparticle distances. The rough surface of SiO_2_ enhances interactions with rubber molecular chains, particularly ENR, forming a bound rubber layer that contributes to reinforcement. However, at higher SiO_2_ concentrations within the bulk TW/ENR matrix, the polar functional groups on the SiO_2_ surface promote filler–filler interactions, leading to agglomeration and a subsequent reduction in reinforcement efficiency [22].

### 3.2. Tensile Properties

**Figure 3:**
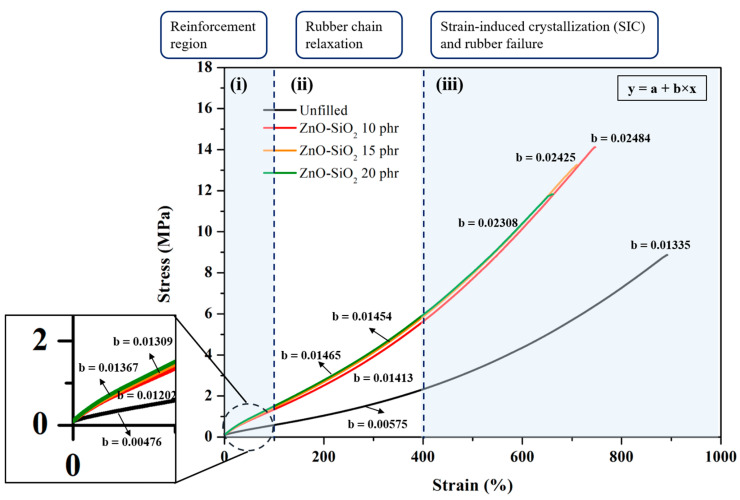
Stress–strain curves of TW/ENR filled with different concentrations of ZnO-SiO_2_ to elucidate crosslinking behavior.

**Figure 4:**
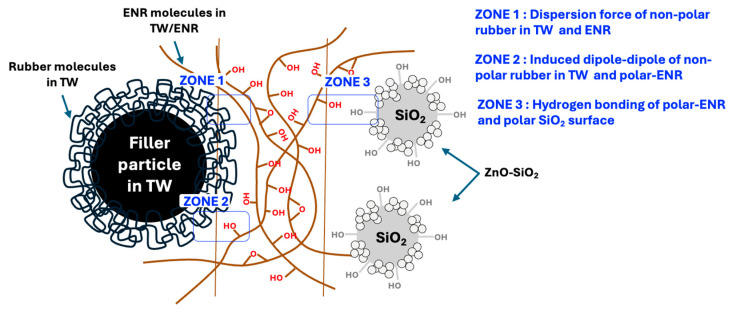
Proposed interaction of ENR with SiO_2_ in ZnO-SiO_2,_ as well as the TW phases separation.

Figure 3 shows the stress–strain plots, performed according to mechanical measurement standards. The formation of crosslinks that provide resistance to breakage, and three distinct regions of the tensile curves, are interpreted as follows:

(i) **Reinforcement region:** Without ZnO-SiO_2_, the blends exhibit poor vulcanization, and their strength arises mainly from chain entanglement contributed by additional ENR. Young’s modulus and the 100% modulus reflect these effects, with both increasing as ZnO-SiO_2_ concentration rises. This behavior confirms an increase in chemical crosslink density, which enhances the blends’ resistance to chain breakage. Notably, the modulus of blends without ZnO-SiO_2_ is very low, indicating that the residual carbon black (CB) in TW does not provide significant reinforcement due to the insufficient diffusion of ENR molecules onto the particle surfaces. Conversely, the addition of ZnO-SiO_2_ facilitates the activation of chemical crosslinking between the remaining TW and un-crosslinked ENR chains. The SiO_2_ particles function as a reinforcing filler, interacting with rubber molecules to prevent blend breakage under applied mechanical deformation [23]. However, at concentrations exceeding 10 phr, no significant improvement is observed due to SiO_2_ particle agglomeration.
(ii) **Rubber chain relaxation:** This region corresponds to the physical interactions between rubber chains and filler surfaces. An increased slope in this region indicates stronger rubber–rubber and rubber–filler interactions, resulting in greater friction among chains during extension. The slope of this region increases with ZnO-SiO_2_ concentrations in the range of 10–20 phr, which can be attributed to the enhanced crosslink density and improved ENR-SiO_2_ interactions.
(iii) **Strain-induced crystallization (SIC) and rubber failure:** SIC occurs as molecular chains align into a crystalline structure during elongation, typically noticeable at approximately 400% strain. Figure 3 shows that the increased crosslink density from ZnO activation enhances the degree of crystallization. Furthermore, hydrogen bonding between ENR molecules and SiO_2_ particles accelerates the alignment of molecular chains along the direction of extension, as depicted in the proposed interaction mechanism in Figure 4 (Zone 3) [23,24]. However, differences in SIC improvement are reflected in the tensile strength and elongation-at-break data presented in Table 3. This difference highlights the need to consider the entire TW/ENR matrix, as shown in Figure 4 (Zones 1 and 2). In the TW proportion of the blends, filler particles with rubber molecular chain absorption are present. Without pyrolysis, rubber chains remain embedded on the filler surfaces as glassy or sticky bound rubber, with only the outer chains interacting with the SiO_2_ surface and ENR molecules [25]. These interactions occur via induced dipole–dipole forces between the SiO_2_ surface and polar groups on ENR molecules (Zone 2), as well as induced dipole–induced dipole interactions on the non-polar regions of ENR (Zone 1) [26]. However, at excessive ZnO-SiO_2_ concentrations, SiO_2_ agglomeration inhibits interactions between the TW and ENR phases, leading to reduced tensile strength and limited elongation at break. It is important to note that ENR chains can also interact with ZnO particles due to the polarity of ZnO, particularly at higher concentrations. However, this interaction primarily occurs during the mixing and compounding stages. During vulcanization, the applied heat facilitates the release of Zn^2+^ ions and ROS from the ZnO surfaces, shifting ZnO’s role to that of an activator for chemical crosslinking. Additionally, the residual ZnO in the rubber matrix continues to release ions, which predominantly contributes to properties such as antibacterial activity. This indicates that the reinforcement efficiency of the blends is primarily associated with the SiO_2_ particles.

### 3.3. Antibacterial Properties and Morphologies

The antibacterial efficiency of the TW/ENR blends is attributed to the release of zinc ions (Zn^2+^) onto the blend surfaces, facilitated by the thickness of the bound rubber layer surrounding the ZnO embedded in TW and the additional ZnO-SiO_2_ at varying concentrations. This occurs because the excess ZnO in both TW and ENR continues to release Zn^2+^ and reactive oxygen species (ROS) onto the rubber surfaces. The ROS react with bacterial cell walls, while Zn^2+^ penetrates the bacterial cells, interacting with their DNA and cytoplasm [27,28,29]. This mechanism, previously described in the literature, highlights the antibacterial activity of ZnO. Based on the crosslinking curves in Figure 1, it is evident that ZnO remains active even though the sulfur has been fully consumed during tire manufacturing. This demonstrates that the antibacterial properties of the blend arise from the positive synergy between residual ZnO in TW and the newly introduced ZnO-SiO_2_.

To investigate the ability of Zn^2+^ and ROS to migrate through the rubber layer covering the ZnO surfaces, a swelling test was conducted to measure the total bound rubber (*ß*) and bound rubber layer thickness (*δ’*) in the blends with increasing ZnO-SiO_2_ concentrations. Blend compounds without accelerators or sulfur were pressed at 160 °C for 5 min, and approximately 2 g of the sample powders were placed in specific sieves and immersed in toluene at room temperature for 7 days. The weight of the blends was recorded, and the *ß* value was calculated using Equation (2), as provided below:

*ß* (%) = (*W_fg_* − *W_f_*)/*W_p_* (2)

where *W_f_* is the approximated weight of the filler in TW received from the remaining ash of the thermogravimetric analysis (TGA) and additional ZnO-SiO_2_ concentrations. The factor *W_p_* is the weight of the rubber, estimated from the TGA and ENR existing in the blends, and *W_fg_* is the weight of the remaining filler with absorbed rubber after toluene extraction. Also, the *δ’* was evaluated through Equation (3) as follows:

*δ’* (*nm*) = (*m*_2_ − *m*_1_(*C_f_*))/*ρ_r_m*_1_(*C_f_*)(*S_f_*) (3)

where *m*_1_ is the mass of the rubber compound before extraction, and *m*_2_ is the mass of the rubber–filler gel consisting of the non-dissolving bound rubber part and the filler. Parameters *C_f_* and *S_f_* are the mass concentration and the specific surface area of fillers in the blends regarding carbon black and SiO_2_, respectively, whereas *ρ_r_* refers to the density of solely ENR matrix.

The results of the *ß* and *δ’* values are summarized in Table 4. It was found that the TW/ENR blend exhibited approximately 20% bound rubber, correlating with the rubber absorption on the filler surfaces in TW and indicating a *δ’* value of 14.12%. This is attributed to the absence of pyrolysis reactions during the reclamation processes. On the other hand, the addition of ZnO-SiO_2_ to the blends increased the *ß* value while reducing the *δ’* value. This behavior is due to the increase in SiO_2_ filler content, which enhances rubber molecule absorption on the rough surface of the filler [30]. Consequently, the *δ’* value tends to decrease significantly. With an increasing ZnO-SiO_2_ content, the highest *ß* value was observed at 10 phr, accompanied by the lowest *δ’* value. This is likely due to the onset of ZnO-SiO_2_ agglomeration within the TW/ENR matrix. This phenomenon highlights the enhanced opportunity for ion movement through the blend’s surfaces, enabling further reactions with bacterial cell walls.

**Figure 5:**
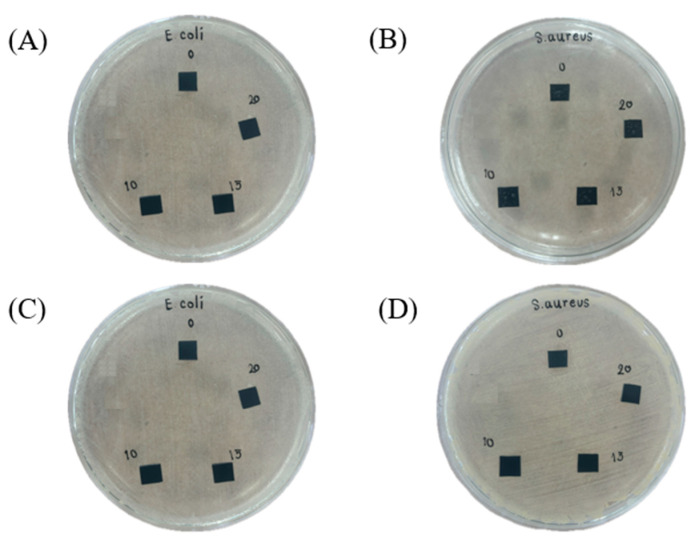
Disk diffusion qualitative measurement of TW/ENR filled with different concentrations of ZnO-SiO_2_ against *Gram-negative E. coli* and *Gram-positive S. aureus* was performed at 8 h (**A**,**B**) and 24 h (**C**,**D**).

The antibacterial efficiency was initially evaluated using qualitative disk diffusion and quantitative cell counting methods under controlled conditions for 8 and 24 h. As shown in Figure 5, the qualitative test revealed no distinct inhibition zone. This suggests that the antibacterial activity, attributed to Zn^2+^ ions and reactive oxygen species (ROS), is likely confined to the surface of the blend, even at ZnO-SiO_2_ concentrations of up to 20 phr, and does not extend to the surrounding areas. This behavior contrasts with the broader diffusion observed with silver (Ag) and copper (Cu) nanoparticles [31,32,33]. Thus, the use of ZnO-SiO_2_ to release Zn^2+^ ions and ROS does not lead to the contamination of adjacent surfaces.

**Figure 6:**
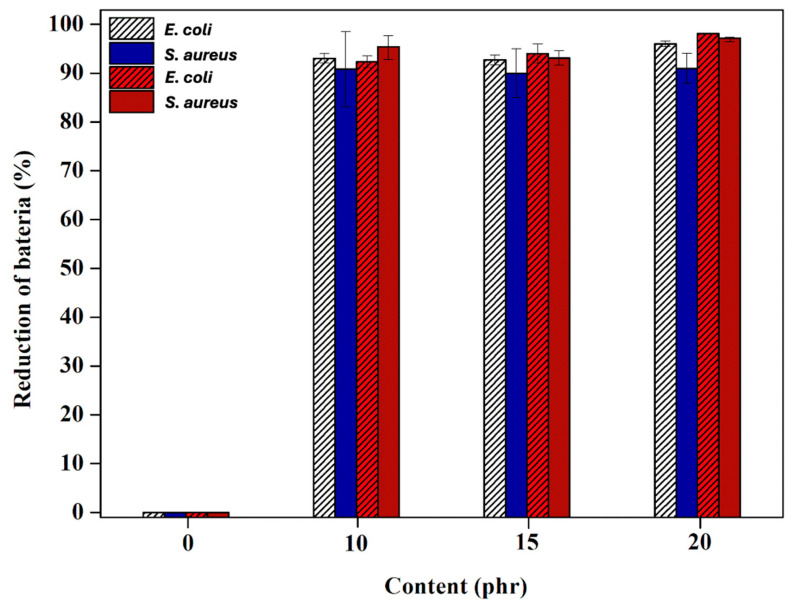
Antibacterial activities of the TW/ENR filled with different concentrations of ZnO-SiO_2_ against *E. coli* and *S. aureus* within 8 h before and after aging at 70 °C for 72 h.

**Figure 7:**
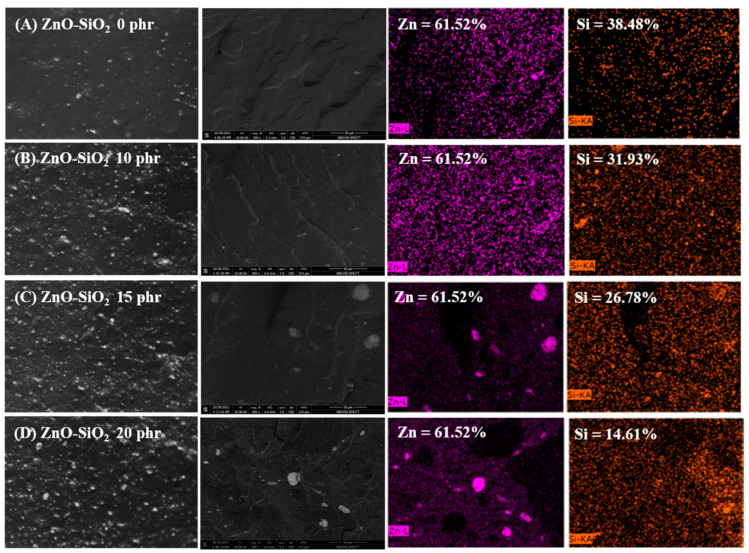
OM and SEM images, along with EDX elemental analysis, of TW/ENR blends filled with different concentrations of ZnO-SiO_2_ after aging for 72 h at 70 °C: blend without ZnO-SiO_2_ (**A**), blends with ZnO-SiO_2_ at concentrations of 10 phr (**B**), 15 phr (**C**), and 20 phr (**D**) together with atom percentage % within TW/ENR matrix.

To evaluate the durability of the antibacterial performance after prolonged use, the blends were subjected to thermal aging at 70 °C for 72 h in a hot air oven, followed by quantitative antibacterial testing. As shown in Figure 6, the antibacterial efficiency remained largely unchanged, except for the TW/ENR blend without ZnO-SiO_2_. This result indicates that the release of Zn^2+^ ions and ROS under photocatalytic conditions is limited in blends containing only ZnO. In contrast, blends with at least 10 phr of ZnO-SiO_2_ maintained their antibacterial activity even under these harsh conditions. Figure 7 illustrates the surface morphologies and elemental composition analyzed using OM and SEM-EDX. As shown in Figure 7A, the TW/ENR blend without ZnO-SiO_2_ exhibits a smooth surface without agglomeration, as ZnO-SiO_2_ is absent from the bulk matrix. However, in the TW/ENR matrix with ZnO alone, the ZnO particles were dispersed unevenly, potentially limiting the release of Zn^2+^ ions and ROS to the surface.

In blends containing 10 phr of ZnO-SiO_2_, the optimal dispersion and distribution of ZnO throughout the TW/ENR matrix was achieved, as seen in Figure 7B. On the other hand, strong agglomeration was observed in blends with 15 or 20 phr of ZnO-SiO_2_, indicating that the excessive SiO_2_ content from ZnO modification led to particle clustering (Figure 7C,D) although a high concentration of Zn elements was found at the blend’s surfaces. This agglomeration not only reduced antibacterial efficiency but also negatively affected the tensile properties, including tensile strength and elongation at break. Based on these findings, the optimal concentration of ZnO-SiO_2_ was identified as 10 phr. At this concentration, ZnO acts effectively as an activator to enhance the antibacterial efficiency of the blends, while SiO_2_ contributes to reinforcement. However, concentrations exceeding 10 phr result in pronounced agglomeration, compromising both mechanical and antibacterial properties.

## 4. Conclusions

The present study explored the effects of incorporating ZnO-SiO_2_ into TW/ENR blends, at a ratio of 50:50 phr, on cure characteristics, mechanical properties, and antibacterial efficiency, with the key findings summarized below:

- The incorporation of ZnO-SiO_2_ significantly enhanced the crosslink density, as indicated by the increased maximum torque and shorter cure times. At the optimal ZnO-SiO_2_ loading of 10 phr, the composite exhibited the fastest crosslinking rate, attributed to the balanced effects of chemical activation and reinforcement. However, higher SiO_2_ concentrations (>10 phr) resulted in filler agglomeration, which negatively impacted the crosslinking efficiency and mechanical properties.
- Tensile properties were enhanced at the optimal concentration of ZnO-SiO_2_ at 10 phr. The enhancements in Young’s modulus and tensile strength were attributed to the synergistic interactions between ENR molecules and SiO_2_ surfaces, as well as strain-induced crystallization, which further reinforced the matrix during extension. However, at concentrations beyond 10 phr, particle agglomeration reduced the reinforcement efficiency, leading to reduced tensile strength and elongation at break.
- ZnO-SiO_2_ contributed significantly to antibacterial activity, achieving up to 99.9% bacterial reduction at 10 phr. This efficiency was attributed to the release of Zn^2+^ ions and reactive oxygen species (ROS), which interact with bacterial cell walls and DNA. Notably, the antibacterial performance remained stable even after heat aging, confirming the durability of the ZnO-SiO_2_-modified blends. However, higher ZnO-SiO_2_ concentrations led to agglomeration, reducing the surface exposure and antibacterial effectiveness.
- The study identified 10 phr as the optimal ZnO-SiO_2_ concentration, offering a balance between mechanical reinforcement, crosslinking efficiency, and antibacterial performance. While ZnO served as an activator and antibacterial agent, SiO_2_ provided mechanical reinforcement through its rough surface interactions with ENR molecules.

These findings highlight the dual functionality of ZnO-SiO_2_ in TW/ENR composites, showcasing its potential applications as a result of enhanced mechanical performance and antibacterial activity. This study lays the foundation for optimizing rubber-based materials for a wide range of applications, including healthcare and engineering. Further investigations into alternative filler designs and advanced processing methods could provide additional opportunities to enhance performance while minimizing agglomeration effects.

## Figures and Tables

**Table 1:** Formulation compositions of TW/ENR blends filled with modified ZnO-SiO_2_.

Ingredients	Content (phr *)
TW/ENR	50/50
Stearic acid	1
ZnO-SiO_2_	0, 10, 15, and 20
MBTS	1
Sulfur	2.5

* phr refers to part per hundred rubber.

**Table 2:** Crosslink properties of TW/ENR blends from compounds to vulcanizates with varying concentrations of the ZnO-SiO_2_ activator.

Formulations	*T_s_*_1_ (min)	*T*_90_ (min)	*M_L_* (d.Nm)	*M_H_* (d.Nm)	*M_H_*–*M_L_* (d.Nm)
Unfilled	1.37	2.18	0.60	5.59	4.98
ZnO-SiO_2_ 10 phr	1.72	4.65	0.77	10.86	10.09
ZnO-SiO_2_ 15 phr	1.77	5.00	0.82	11.48	10.60
ZnO-SiO_2_ 20 phr	1.81	5.28	0.89	12.00	11.11

**Table 3:** Mechanical properties of TW/ENR blend with different modified ZnO-SiO_2_ loading.

Formulations	100% Modulus (MPa)	300% Modulus (MPa)	Tensile Strength (MPa)	Elongation at Break (%)
Unfilled	0.60 ± 0.02	1.64 ± 0.04	8.79 ± 0.15	879.72 ± 22.09
ZnO-SiO_2_ 10 phr	1.35 ± 0.02	3.91 ± 0.05	14.24 ± 0.16	749.77 ± 22.86
ZnO-SiO_2_ 15 phr	1.45 ± 0.01	4.16 ± 0.03	13.59 ± 0.19	715.51 ± 25.22
ZnO-SiO_2_ 20 phr	1.50 ± 0.04	4.25 ± 0.03	12.34 ± 0.38	668.86 ± 37.80

**Table 4:** Results of bound rubber and bound rubber layer thickness of the TW/ENR blends for elucidating the Zn^2+^ and ROS ions diffusion.

Formulations	*ß* (%)	*δ’* (nm)
Unfilled	20.43 ± 0.13	14.12 ± 0.11
ZnO-SiO_2_ 10 phr	30.44 ± 1.05	2.99± 0.01
ZnO-SiO_2_ 15 phr	29.78 ± 1.12	4.34 ± 0.13
ZnO-SiO_2_ 20 phr	27.93 ± 1.78	4.95 ± 0.02

## Data Availability

All the data are available from the authors and can be provided on request.
